# Genomic analysis of *Sparus aurata* reveals the evolutionary dynamics of sex-biased genes in a sequential hermaphrodite fish

**DOI:** 10.1038/s42003-018-0122-7

**Published:** 2018-08-17

**Authors:** Marianna Pauletto, Tereza Manousaki, Serena Ferraresso, Massimiliano Babbucci, Alexandros Tsakogiannis, Bruno Louro, Nicola Vitulo, Viet Ha Quoc, Roberta Carraro, Daniela Bertotto, Rafaella Franch, Francesco Maroso, Muhammad L. Aslam, Anna K. Sonesson, Barbara Simionati, Giorgio Malacrida, Alessandro Cestaro, Stefano Caberlotto, Elena Sarropoulou, Costantinos C. Mylonas, Deborah M. Power, Tomaso Patarnello, Adelino V. M. Canario, Costas Tsigenopoulos, Luca Bargelloni

**Affiliations:** 1 Department of Comparative Biomedicine and Food Science, University of Padova, viale dell’Università, 16 35020 Legnaro, Italy; 2 Institute of Marine Biology, Biotechnology and Aquaculture ó, Hellenic Centre for Marine Research, Thalassocosmos, Former US Base at Gournes, 715 00 Heraklion, Greece; 3 CCMAR-Centro de Ciências do Mar, University of Algarve, Campus de Gambelas, 8005-139 Faro, Portugal; 4 Department of Biotechnology, University of Verona, Strada Le Grazie 15, 37134 Verona, Italy; 5 Nofima, P.O. Box 210, N-1431 Ås, Norway; 6 BMR Genomics, Via Redipuglia 21a, Padova, Italy; 7 Research and Innovation Centre, Fondazione Edmund Mach, via Edmund Mach 1, 38010 San Michele all’Adige, Trento Italy; 8 Valle Cà Zuliani Società Agricola Srl, Via Timavo 76, 34074 Monfalcone, Gorizia Italy

## Abstract

Sexual dimorphism is a fascinating subject in evolutionary biology and mostly results from sex-biased expression of genes, which have been shown to evolve faster in gonochoristic species. We report here genome and sex-specific transcriptome sequencing of *Sparus aurata*, a sequential hermaphrodite fish. Evolutionary comparative analysis reveals that sex-biased genes in *S. aurata* are similar in number and function, but evolved following strikingly divergent patterns compared with gonochoristic species, showing overall slower rates because of stronger functional constraints. Fast evolution is observed only for highly ovary-biased genes due to female-specific patterns of selection that are related to the peculiar reproduction mode of *S. aurata*, first maturing as male, then as female. To our knowledge, these findings represent the first genome-wide analysis on sex-biased loci in a hermaphrodite vertebrate species, demonstrating how having two sexes in the same individual profoundly affects the fate of a large set of evolutionarily relevant genes.

## Abstract (web summary)

Marianna Pauletto, Tereza Manousaki et al. present the genome sequence of *Sparus aurata*, a sequential hermaphroditic fish. Comparative analysis with gonochoristic species shows that sex-biased genes generally evolve more slowly in *S. aurata* due to stronger functional constraints.

## Introduction

How two separate sexes evolve using nearly the same genetic information is one of the most fascinating subjects in evolutionary biology^1^. Female and male phenotypes are often quite different and such sexual dimorphism is largely due to differential regulation of shared genes, as there are few completely sex-specific loci^2,3^. Sex-biased gene expression also contributes to resolve antagonistic conflicts between the sexes^1,3^. Recent advances in genomics have offered the opportunity to study sex-biased (SB) gene expression and the evolutionary dynamics of SB genes in non-model species^4^. In nearly all cases, male-biased genes show higher rates of protein-coding sequence evolution, although exceptions have been reported^4,5^. In teleost fishes, a recent study on zebrafish *Danio rerio* (with a polygenic sex determination system) and three-spine stickle back *Gasterosteus aculeatus* (with a XY sex determination system) reported faster evolution for male-biased genes^6^ and similar evidence was also found in the ocellated wrasse, *Symphodus ocellatus*^7^.

Although the evolution of SB genes has been studied in several gonochoristic vertebrates (i.e., having separate sexes, as opposed to hermaphroditic species) and invertebrates, little is known for hermaphrodite species. This is quite unfortunate, because SB gene expression is undoubtedly an important feature in hermaphrodites, as males and females share identical genetic information and their peculiar reproductive system might substantially influence the evolution of SB genes. Indeed, sexual conflict is peculiar in hermaphrodites compared with gonochoristic species^8,9^. To our knowledge, the evolution of SB genes has been analysed in only two hermaphrodite organisms, in the androdioecius (i.e., having both hermaphroditic and male individuals) nematode *Caenorhabditis elegans* and in the fungus *Neurospora crassa*. In *C*. *elegans*, faster evolution of genes involved in spermatogenesis was observed^10^, whereas in *N*. *crassa* female-biased genes evolved faster^11^. It is, therefore, of interest to extend the analysis to other hermaphrodites, including more complex organisms such as the vertebrates. Unique among the vertebrates, the bony fishes (Superorder Teleostei) show both simultaneous and sequential forms of hermaphroditism^12^, making them an ideal subject of investigation.

The gilthead sea bream (*Sparus aurata* Linnaeus, 1758) is a temperate marine teleost of great relevance for marine aquaculture^13^ and its biology is well characterized with special focus on immunology, reproductive physiology, and nutrition. A unique and challenging feature for aquaculture of this species is that it is a sequential hermaphrodite. In larvae, ovaries start to differentiate but are replaced by the testes so that in the first reproductive cycle *S*. *aurata* mature first as males at the age of 2 years old^14^. In the following cycles and depending on social factors, the testis regress and in some males a functional ovary develops. A direct consequence is that females are always larger than males. *S*. *aurata* belongs to the economically important Sparidae family, which is largely composed of either simultaneous or sequential hermaphrodite species, both protandric and protogynous, although gonochoristic species are also present^12,15^. This makes this family of fishes a rather unique opportunity to investigate hermaphroditism and the evolution of SB genes. In this context, we report the first annotated genome, to our knowledge, of a protandric sparid species. Using comparative genomic and transcriptomic approaches, we demonstrate for the first time in a hermaphrodite vertebrate species that the evolutionary patterns of SB genes are highly divergent from what is observed in gonochoristic species.

## Results

### The gilthead sea bream genome

To sequence the *S*. *aurata* genome, a combined approach was followed:^16^ Illumina paired-end sequencing was performed on DNA extracted from a double haploid (fully homozygous) individual to construct a first draft genome assembly and PacBio long reads were used to close gaps. The N50 and L50 statistics were 37,409 and 5476 for the scaffolds, and 35,872 and 5750 for the broken scaffolds (i.e., contigs, see Supplementary Table 1). The resulting scaffolds were further ordered and oriented by anchoring them to three high-density genetic linkage maps. The combination of multiple maps greatly improved the accuracy of the assembly. The assembled *S*. *aurata* genome consists of 24 super-scaffolds (i.e., chromosomes, including *N*’s between the ordered contigs), corresponding to the number of known linkage groups, and 34,623 shorter scaffolds. The quality of the genome assessed with BUSCO (Supplementary Table 2), highlighted a percentage of 90.8% complete BUSCO groups (88.9% in single copy).

Genome annotation was based on similarity and experimental evidence from RNA expression was obtained using existing or newly generated RNA sequencing (RNA-seq) data. The total number of estimated genes was 30,454, in line with the number of genes identified in the *Dicentrarchus labrax* genome (Supplementary Table 3). The *S*. *aurata* genome can be accessed through a dedicated Genome browser (http://biocluster.her.hcmr.gr/myGenomeBrowser?portalname = Saurata_v1).

### Homology, phylogeny, and gene family expansions

**Fig. 1:**
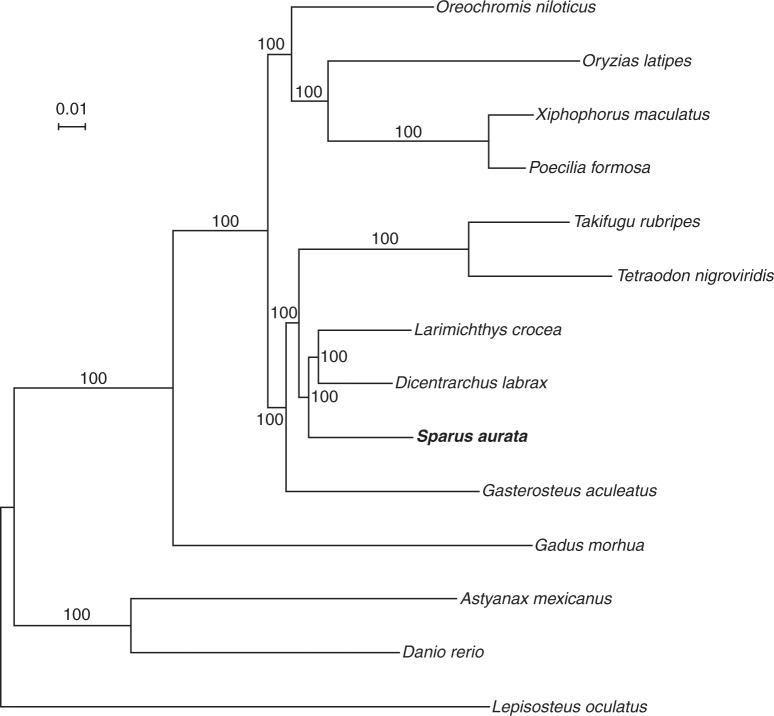
Phylogenetic tree. Phylogenetic relationships of *S. aurata* and other teleost fishes. Numbers refer to bootstrap values. The tree was constructed using 762,730 amino acid sites from 2032 orthologous genes, using maximum likelihood methodology under JTT + F + Γ model

A phylogenetic tree including selected teleost species with a high-quality draft genome was constructed using OMA, to establish homology relationships and to provide a robust evolutionary framework for subsequent analysis (Fig. 1)^17^. The constructed phylogeny overall agrees with the known relationships among the analysed teleost species and indicates that a comparison with the European sea bass as sister group and three-spined stickle back as outgroup is the most appropriate for the analysis of branch-specific evolutionary rates.

OMA results, which are quite conservative but highly accurate^18^, were used to identify protein-coding gene families and to estimate protein family expansions and contractions, relative to other teleost genomes (Supplementary Data 1). Although such comparison should be taken with caution as the quality of genome assembly is variable across the species used in this analysis, which might inflate the number of putative duplicated genes, a comparison within the same genome should not be substantially affected by such a bias. We thus compared the number of expanded/contracted gene families and the number of gene copies within families between female-biased and male-biased genes (see below) in *S*. *aurata*. Among expanded protein families, 68 were identified to have at least one ovary-specific copy, which was not significantly different (Fisher’s exact test *p* > 0.1) compared with the 80 showing at least one testis-specific family member. Three contracted protein families were found in female-biased genes and eight in male-biased ones (Fisher’s exact test *p* > 0.3). There were 44 protein families that contained both female- and male-biased genes. Although the number of expanded/contracted protein families was similar, when comparing only the gonad SB members of the expanded/contracted families, we found that the number of their male-biased gene members (458) was significantly higher (Fisher’s exact test, *p*-value = 2.2 × 10^−16^) than the number of their female-biased ones (212). Three gene families (two expanded and one contracted) included both gonad and brain SB members (Supplementary Data 2) including the contracted major histocompatibility complex class-I-related gene family. Finally, three expanded families included only male-biased genes (without any unbiased (UB) family member).

### SB brain and gonad transcriptome analysis

Analysis of male- and female-biased transcripts in the brain of adult individuals identified only a small set of 66 differentially expressed brain genes (Supplementary Data 3), using the significance threshold most commonly adopted for identifying SB genes (log_2_ fold change (FC) > 1, false discovery rate FDR < 0.05)^4^. These results are consistent with previous observations in teleosts^19–22^ and in other species (reviewed in ref. ^4^). Among the 24 genes overexpressed in the male brain, *isotocin-neurophysin IT 1-like* (Sa_52920.4) and *vasotocin-neurophysin VT 1-like* (Sa_28561.3) are putatively associated with male social behaviors. The most interesting female-biased gene was Sa_20946.1. No known protein domains could be identified in open reading frames within this transcript. However, Sa_20946.1 appeared to have high homology with *reproduction regulator 2*, a gene that was reported to be expressed in the hypothalamus and associated with sex reversal in another sequential hermaphrodite teleost, the orange-spotted grouper *Epinephelus coioides*^23^. Similar brain-specific and SB expression between two sequential hermaphrodite fish suggests a potentially conserved role in sex reversal for this poorly characterized transcript and warrants for further analysis. In contrast, thousands of testis (8524) and ovary (7854) genes were significantly overexpressed (log_2_ FC > 1; FDR < 0.05) (Supplementary Data 3). The number of differentially expressed genes is consistent with similar studies that have been carried out in fish^20,21^. Functional annotation revealed significant enrichment (gene count ≥ 2; ease value > 0.1) for cilium-related biological processes in male-biased genes and rRNA, tRNA, and mRNA (nuclear and mitochondrial) processing pathways in female-biased genes (Supplementary Data 4). This is consistent with the sperm-producing role of the testis and the egg-producing function of the ovary, and is similar to what has been reported in other species (e.g., see ref. ^24^). A strong expression bias (log_2_ FC ≥ 3) was significantly more frequent (*χ*^2^-, *p*-value < 0.0001) in male-biased genes (43%) than in female ones (28%), which was also in agreement with previously evidence^6,24^.

It has long been known that genes with co-regulated expression tend to be clustered in eukaryote genomes^25–27^. However, differentially expressed gonadal genes showed only limited evidence of clustering in the *S*. *aurata* genome, as revealed using two independent tests (Supplementary Data 5). Testis-biased genes appeared to be significantly clustered only on chr18 (Fisher’s exact test, *p*-value = 0.02) and ovarian-biased genes on chr23 (Fisher’s exact test, *p*-value = 0.01). Chr2 and chr24 showed significant clustering (Fisher’s exact test, *p*-value = 0.02 for Chr2 and *p*-value = 0.04 for Chr24) when the analysis was limited to genes with a strong male bias (log_2_ FC ≥ 3) and only chr1 clustered genes with a strong female bias (Fisher’s exact test, *p*-value = 0.02). Functional annotation of significantly clustered (Fisher’s exact test, *p*-value < 0.05) SB genes did not identify any significant Gene Ontology (GO) term, except for male-biased genes clustering on chr18, where the GO term *Regulation of transcription, DNA templated* (GO:0006385) was enriched at nominal *p*-value = 0.082 (FDR = 0.4).

When looking at the genes belonging to a family and single-copy genes, we found that the percentage of single-copy genes found among female-biased genes was significantly higher than that found in UB genes (Fisher’s exact test = 0.00001) (Supplementary Table 4). Although it is not immediately clear how to interpret these results, a possible consideration is that single-copy genes tend to be more pleiotropic than genes belonging to gene families; therefore, female-biased genes might be less specialized than UB and male-biased ones.

### Comparative transcriptomic analysis of SB genes

**Fig. 2:**
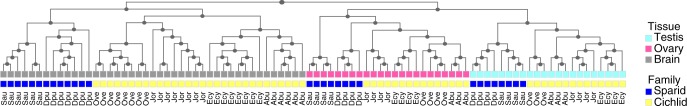
Hierarchical clustering analysis. Clustering based on RNA-seq (gonads and brains) analysed in six species. Sau *S. aurata*, Dpu *D. puntazzo*, Ove *O. ventralis*, Jor *J. ornatus*, Ecy *E. cyanostictus*, Abu: *A. burtoni*

To further evaluate SB gene expression in *S*. *aurata*, a comparative transcriptomics approach was deployed^28^ using the analysis of previously published brain and gonad RNA-seq data from *Diplodus puntazzo*, another sparid^22^, and from four cichlids *Eretmodus cyanostictus*, *Astatotilapia burtoni, Ophthalmotilapia ventralis* and *Julidochromis ornatus*^21^. In order to compare gene expression data, one-to-one strict orthology relations between *S*. *aurata* and *Oreochromis niloticus* were retrieved with OMA and resulted in a total of 13,873 one-to-one orthologous genes. These genes were assumed to have a one-to-one orthology relation also with *D*. *puntazzo* and the four other cichlids species here considered. The transcriptional profiles of the 13,873 orthologous genes in sparids and cichlids clustered first by organ (respectively, brain, testis, and ovary) and then by species (Fig. 2), as previously observed for tissue-specific expression of protein-coding genes in several other mammals species^29^. The number of SB genes was extremely limited in the brain across the six species (range 0–26) and no shared genes were identified even when *A*. *burtoni*, a species with no SB genes in the brain, was excluded. As expected, several thousand genes showed a significant (log_2_ FC > 1, FDR < 0.05) sex bias in the gonads of all species, with comparable numbers between sparids and cichlids. A core of 765 female-biased and 970 male-biased genes was conserved across species, i.e., between the two fish lineages, which diverged ~100 million years ago^30^ (Fig. 1). The limited percentage (~20%) of genes with SB expression across the six different species is similar to that observed in birds within a comparable evolutionary time frame and confirms the rapid turnover of SB genes among species^24^.

### Evolutionary dynamics of SB genes

**Fig. 3:**
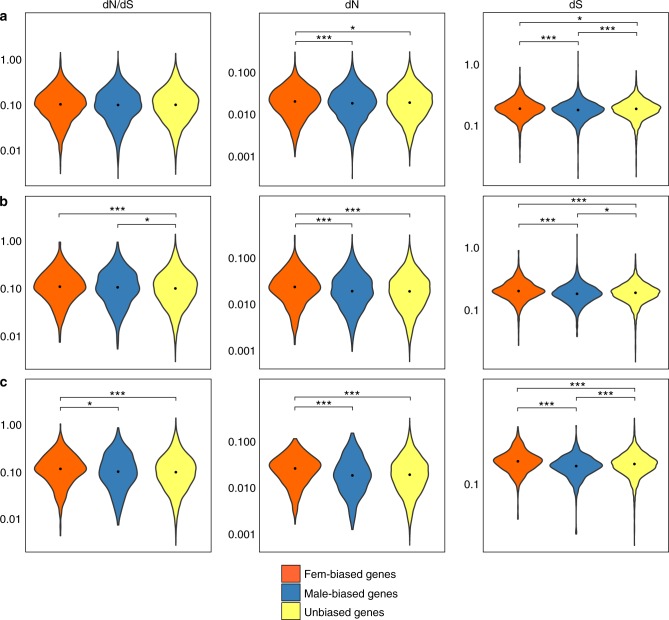
Synonymous and non-synonymous substitution rates. Violin plots describing the distribution of dN, dS, and dN/dS values calculated on sex-biased genes (**a**), sex-biased genes with a log_2_ FC > 3 (**b**), and sex-biased genes conserved in the six species analysed in this study (**c**). Asterisks indicate statistical significance between groups (pairwise Wilcoxon’s rank-sum test: ***p-value < 0.001; **p-value < 0.01; *p-value < 0.05). The black point indicates the median value

Using a well-established maximum likelihood approach (e.g., see refs. ^6,7^.), synonymous (dS) and non-synonymous substitution (dN) rates were specifically estimated for the *S*. *aurata* lineage, through a comparison with *D*. *labrax* and *G*. *aculeatus*. At variance with what has been reported in nearly all species investigated so far, including teleost fish^6,7^, in *S*. *aurata* female- and male-biased genes had similar evolutionary rates to UB genes (Fig. 3). To further investigate such discordant evidence in *S*. *aurata*, we applied the same approach and pipeline to another teleost species, the Nile tilapia, for which high-quality genome sequence and SB transcriptome data were available^31^. The obtained results (Supplementary Data 6) for tilapia were in complete agreement with evidence reported for other teleosts. Male-biased genes and, to a lesser extent, female-biased ones evolved faster than UB genes.

To start dissecting we restricted the analysis to genes showing stronger sex bias (log_2_ FC ≥ 3, Fem-FC3 and Male-FC3), considering that highly biased genes, especially male-biased, are expected to have evolved faster^6,7^. In parallel, as the assignment of SB genes may be affected by species-specific factors (e.g., developmental stage, size, and maturity)^32^, we analysed only those genes that are consistently SB in the six sparid and cichlid species (Fem-6-SP and Male-6-SP), i.e., in evolutionarily distant lineages (Fig. 1)^30^. Surprisingly, female-biased genes (Fem-FC3 and Fem-6-SP) had significantly higher dN/dS rates compared with UB ones (*p*-value = 2.876 × 10^−04^ for Fem-FC3 and *p*-value = 2.725 × 10^−05^ for Fem-6-SP, significant after Bonferroni correction), whereas Male-FC3 genes had only a marginally significant faster rate (*p-*value = 1.558 × 10^−02^, not significant after Bonferroni correction) (Fig. 3). Closer inspection of dN and dS results revealed that both synonymous and non-synonymous substitutions accumulated significantly faster in Fem-FC3 and Fem-6-SP (as concerning dN results, *p*-value = 4.353 × 10^−10^ for Fem-FC3, *p*-value = 3.467 × 10^−11^ for Fem-6-SP, whereas for dS results *p*-value = 7.169 × 10^−10^ for Fem-FC3, *p*-value = 9.169 × 10^−09^ for Fem-6-SP, all significant after Bonferroni correction; Fig. 3). Silent substitutions are generally considered neutral and should have similar fixation rates across genes in the same species. However, it is increasingly evident that mutations at synonymous sites might affect translation efficiency, splicing control elements, microRNA binding, and mRNA stability, making them well visible to natural selection^33^. In fact, silent mutations have been reported to cause several genetic diseases^34^ and it has been calculated that 5–10% of genes in the human genome might contain a region where synonymous mutations have negative effects^35^.

**Fig. 4:**
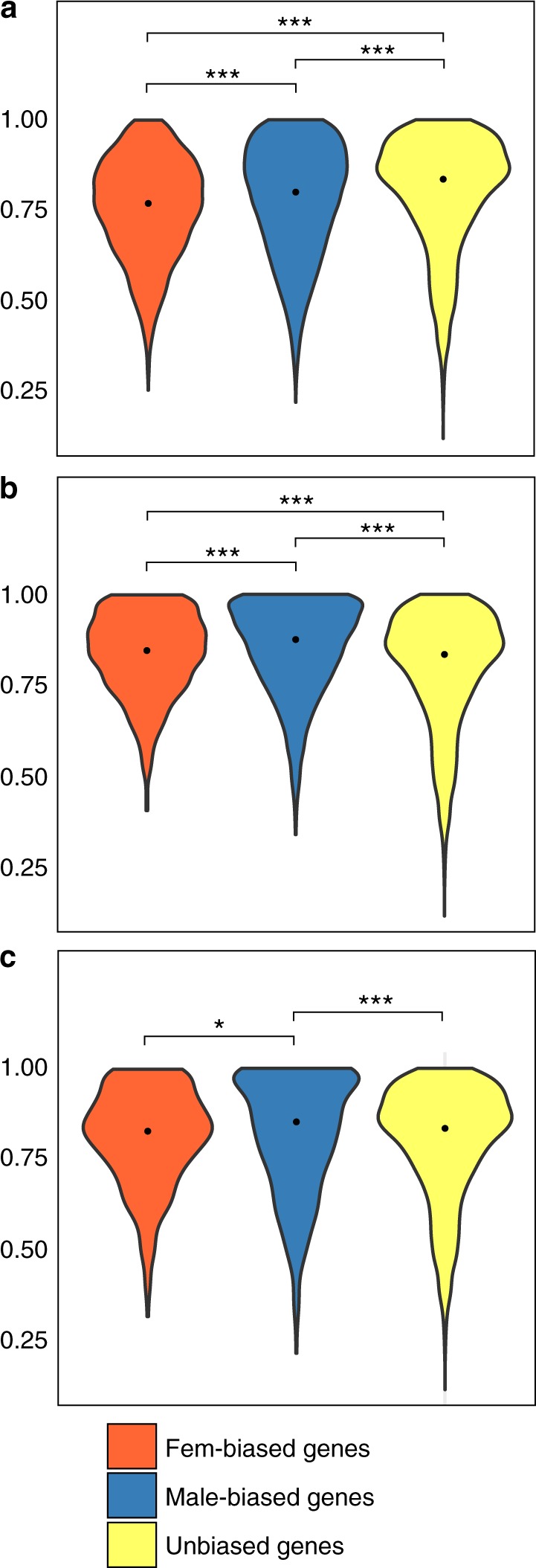
Tissue specificity (*τ*) of sea bream genes. Violin plots describing the distributions of *τ*-values calculated on sex-biased genes (**a**), sex-biased genes with a log_2_ FC > 3 (**b**), and sex-biased genes conserved in the six species analysed in this study (**c**). Tissue specificity was calculated on the mean normalized expression (TMM-normalized log_2_ cpm) evaluated in whole larvae, ovary, testis, brain, gut, heart, liver, skeletal muscle, and spleen. Asterisks indicate statistical significance between groups (pairwise Wilcoxon’s rank-sum test: ***p-value < 0.001; **p-value < 0.01; *p-value < 0.05). The black point indicates the median value

In vertebrates, differential evolutionary rates of SB genes were associated with expression breadth^4,6,36^. In fact, SB genes, especially male-biased genes, have higher tissue specificity than UB genes. Tissue specificity, usually estimated using the *τ*-index^37^, is considered a reliable proxy for limited pleiotropy. Pleiotropy is known to restrict gene evolution, imposing stricter functional constraints on pleiotropic genes^38^. We thus compared *τ* between SB and UB genes. As observed in other species, in *S*. *aurata* male-biased genes appeared more tissue-specific than female-biased ones, and strongly biased (Fem-FC3 and Male-FC3) or conserved SB genes (Fem-6-SP and Male-6-Sp) had narrower expression breadth. However, at variance with previous reports, UB genes were generally more tissue-specific than SB genes (Fig. 4 and Supplementary Figure 1).

Such evidence might explain why evolutionary rates in protein-coding sequences were not significantly different between SB and UB genes. However, *τ*-values do not fully account for the results obtained for either conserved SB (Male-6-SP and Fem-6-SP) or strongly biased genes (Male-FC3 and Fem-FC3), because male-biased genes have a higher *τ*, but lower dN/dS values (Fig. 3).

**Fig. 5:**
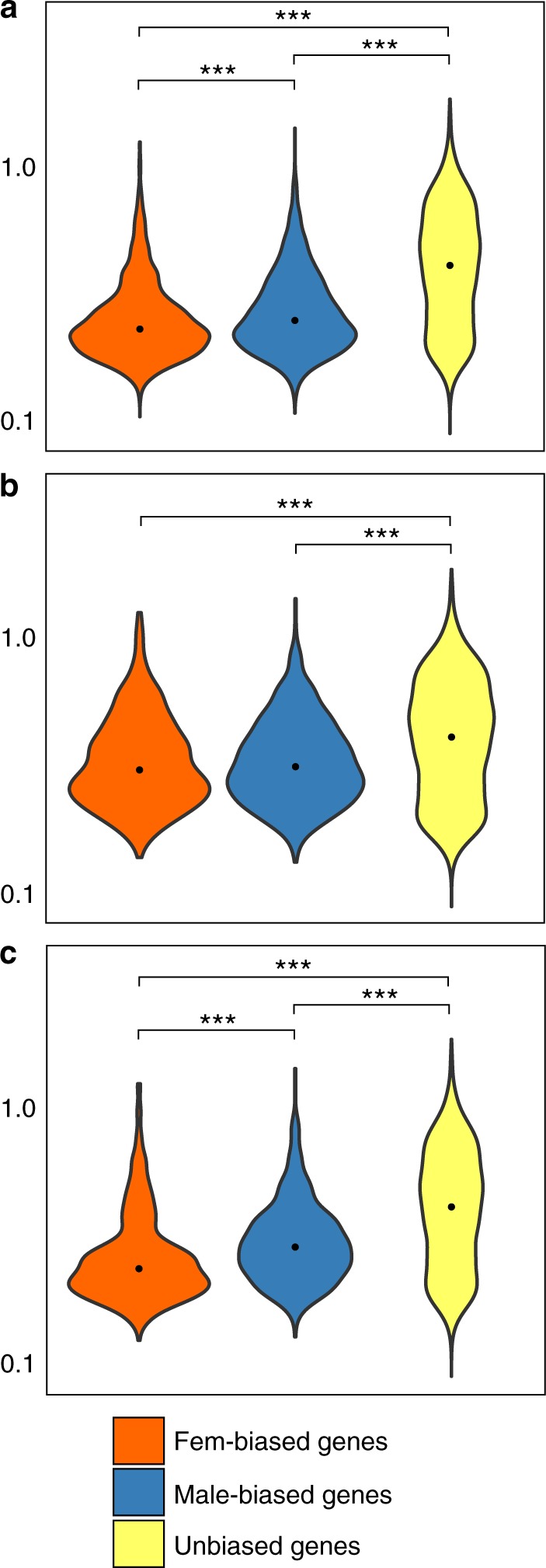
Coefficient of variation (CVE) of sea bream genes. Violin plots describing the distributions of CVE values calculated on sex-biased genes (**a**), sex-biased genes with a FC > 3 (**b**), and sex-biased genes conserved in the six species analysed in this study (**c**). CVE values across the different sets of *S. aurata* unbiased and sex-biased genes were estimated across different tissues and developmental stages. Asterisks indicate statistical significance between groups (pairwise Wilcoxon’s rank-sum test: ***p-value < 0.001; **p-value < 0.01; *p-value < 0.05). The black point indicates the median value

Another variable that has been proposed to correlate with rates of protein-coding gene evolution is the coefficient of variation in gene expression (CVE) across individual samples^39^, with higher CVE values associated with faster evolutionary rates. We estimated the CVE for all *S*. *aurata* genes in the same dataset across different tissues and developmental stages. CVE values across the different sets of SB genes (Fig. 5) are in agreement with the pattern obtained for gene expression breadth (Fig. 4), but did not fully match with the observed rates of sequence evolution (Fig. 3).

**Fig. 6:**
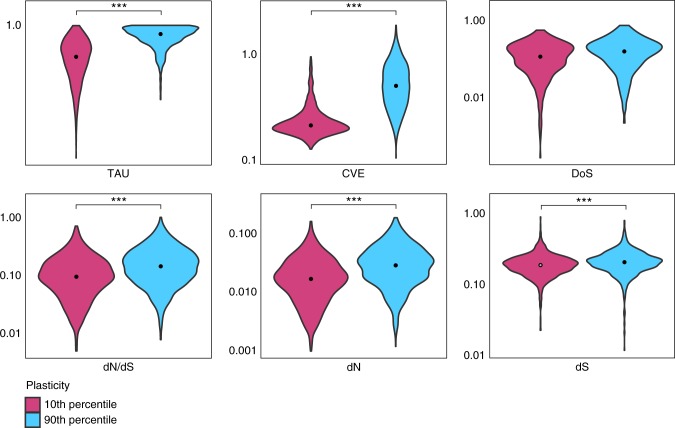
Relation between *τ*, CVE, DoS, evolutionary rates and plasticity (*π*). Violin plots describing the distributions of *τ*, CVE, DoS, dN/dS, dN, and dS values on highly plastic genes and constitutively expressed genes (either in the ≤ 10% or ≥ 90% percentiles based on *π*-values). Gene expression plasticity was quantified across larvae at three stages of development exposed to chronic stress (Supplementary Data 9). Asterisks indicate statistical significance between groups (pairwise Wilcoxon’s rank-sum test: ***p-value < 0.001; **p-value < 0.01; *p-value < 0.05). The black point indicates the median value of each distribution

As it could be possible that our data do not fully conform to the expected association between expression bias, dN, dS, *τ*, and CVE, we decided to test differently such association on the same sequence and expression data, but in a conceptual framework that is not dependent on sex. Genes involved in phenotypic plasticity, i.e., expressed only under specific conditions, are supposed to be more tissue-specific, show greater CVE, and evolve faster than constitutively expressed genes^40^. We used published RNA-seq experiments of the response to environmental stress in *S*. *aurata* larvae at three different developmental stages^41^ to calculate the plasticity index *π*^40^ for each gene. Comparing highly plastic and constitutively expressed genes (979 genes, either in the ≤ 10% or ≥ 90% quantiles based on *π*-values), the association between *π*, *τ*, CVE, and evolutionary rates appeared clearly evident (Fig. 6).

Plastic (stress-responsive) genes had higher tissue specificity, CVE, dN/dS, dN, and dS, in agreement with theoretical expectations, suggesting that *S*. *aurata* sequence and expression data fully conform to the evolutionary model where tissue specificity, variation in gene expression, and conditional expression, should predict evolutionary rates. Therefore, the evidence obtained for dN, dS, and dN/dS in *S*. *aurata* SB genes cannot be explained just based on tissue specificity and variation of gene expression, and it is likely that additional factors related to sex plasticity and reproduction in *S*. *aurata* are involved in determining such evidence.

In fact, two main exceptions emerge from the results presented here. First, the expression breadth is broader than expected^3,6^ in male- and female-biased genes. It has been proposed that intrinsic limits exist for SB regulation of gene expression^1,3^, because males and females share nearly the same genome. It is possible that in *S*. *aurata*, additional constraints are imposed on gene expression given that the same individual first matures as a male and subsequently changes into a female using exactly the same genome. Comparative genomic analysis with other gonochoristic and hermaphrodite fish species should help testing such hypothesis. In a first attempt to address this issue, we aligned the *S*. *aurata* genome against eight draft genome sequences from different teleosts, including two additional protandric sequential hermaphrodites, the Asian sea bass *Lates calcarifer*, and the Asian swamp eel *Monopterus albus*. We searched for highly conserved non-coding elements (CNEs) (200 bp window with at least 80% similarity) and found that Asian sea bass, Asian swamp eel, and *S*. *aurata* shared 26,093 CNEs. This number might be inflated by the limited phylogenetic divergence between the three species, as they all belong to the Acanthomorpha, a teleost lineage that diverged ~150 million years ago^30^. It might also be possible that part of the identified CNEs are actually untranslated transcribed (UTR) regions that have not been properly annotated. Nonetheless, nearly 40% of these CNEs (9904) were present only in the 3 hermaphrodite genomes (Supplementary Data 7). Although the precise role of CNEs remains elusive, the general consensus is that they are involved in gene regulation^42^. They appear to be organized in genomic regulatory blocks, also working at long distance within topologically associated domains^43^, which makes it difficult to directly associate CNEs with the regulation of individual genes. However, the overall evidence of a large fraction of CNEs unique to hermaphrodite fish genomes suggests that these regions might have distinctive roles depending on the biology of the species.

The second unexpected observation was that faster evolutionary rates were observed for strongly biased or conserved female-biased genes, but not for male-biased genes. As already mentioned, the opposite behavior of female- and male-biased genes cannot apparently be explained by patterns of gene expression (*τ* and CVE). Genomic organization for both sets of genes was similar, with near absence of significant clustering (Fisher’s exact test, *p*-value > 0.01 in all the significant tests) in the genome (Supplementary Data 5). The presence of expanded gene families, which are supposed to provide the opportunity for evolving novel functions in duplicated genes (neofunctionalization) or relaxing pleiotropic constraints (subfunctionalization), was not different between female- and male-biased genes. A larger number of duplicated gene copies, which should also allow for greater evolvability, was present in male-biased genes. We should then expect faster evolution in male-biased genes, yet we observe the opposite.

Very few examples of higher rates of evolution of female-biased genes have been reported so far. In birds, it was observed that female-biased genes in early developmental stages evolved faster and were different from the SB genes observed in mature females. This suggests that sex-specific selection pressure varies along the ontogenetic axis as a function of the different biology of male and female reproduction^32^. In *S*. *aurata* and in the majority of species where SB genes have been studied, however, female-biased genes have been identified only in mature females^6,7^. Indeed, genes that are consistently overexpressed in mature female gonads in the six teleost species analysed (Fem-6-SP) also had faster evolutionary rates in *S*. *aurata*.

Faster evolution of female-biased genes has also been reported in the hermaphrodite fungus *N*. *crassa* and such evidence was ascribed, at least in part, to positive selection as a result of female-female competition during mating^11^. Two recent studies on mosquitos also found higher dN/dS rates in female-biased genes^44,45^. Such evidence in *Anopheles* malaria mosquitoes was proposed to result from positive selection of genes that are female-biased and have a role in blood feeding^44^. In *Aedes aegypti*, sexual selection acting on ovary-biased genes was hypothesized^45^. In both studies, reduced male–male competition was suggested to account for the observed lower evolutionary rate of male-biased genes.

It is possible that faster evolution of female-biased genes in *S*. *aurata* was caused by positive selection due to strong female-female competition as proposed for *Anopheles* spp, *A*. *aegypti*, and *N*. *crassa*. Reproduction in *S*. *aurata* occurs in mass-spawning events, where several males and females release sperms and eggs, a situation where same-sex competition is likely. A similar scenario has been proposed to explain faster rates in both male- and female-biased genes in other teleosts^6^. However, it is not clear why accelerated evolution for male-biased genes was not observed in *S*. *aurata*. In fact, male–male competition is expected to be especially high in protandric sequential hermaphrodites^12^.

Divergence of protein-coding sequences between species (dN/dS) might reveal patterns of faster evolution, but it is generally not sufficient to distinguish between adaptive evolution and other factors, such as relaxed purifying selection. Statistical tests that compare interspecific divergence with intraspecific genetic variation might help understanding the nature of selection on coding sequences. We estimated the neutrality index (NI)^46^ using an UB estimator^47^. Values of NI > 1 indicate an excess of non-synonymous polymorphisms, which is expected when slightly deleterious mutations are present, whereas NI < 1 suggests an excess of non-synonymous divergence, as expected under positive selection^47^. All sets of genes, female-biased, male-biased, and UB had NI values > 1 (range 1.25–1.42). To obtain point (gene-by-gene) estimates of NI, we used a second statistic, Direction of Selection (DoS)^47^. The majority of genes in all gene sets had DoS < 0, which means that non-synonymous polymorphism was higher than non-synonymous divergence (see Supplementary Data 8); however, no significant differences (Wilcoxon’s rank-sum test, *p*-value > 0.1) were observed between male- and female-biased gene sets. Overall, these results indicate that purifying selection was relatively more important than adaptive selection in the evolution of *S*. *aurata* genes, but they do not appear to be conclusive on their role on the evolutionary dynamics of SB genes. There are two possible reasons for this. First, NI and DoS statistics assume that silent mutations are neutral, which does not seem to be the case here. Second, data on intraspecific variation in *S*. *aurata* were limited to a small number of farmed individuals, which are unlikely to represent natural genetic variation in the species.

In any case, neither positive selection in relation to same-sex competition nor relaxed purifying selection associated with reduced functional constraints (*τ* and CVE) appear to convincingly explain the different evolutionary rates between female- and male-biased genes. There is an additional hypothesis that has been invoked to account for faster evolution in SB or other “condition”-biased genes. Genes that are expressed only in a fraction of individuals are supposed to experience weaker purifying selection than constitutively expressed ones because of lower effective population size (Ne) for conditionally expressed genes^48,49^. In theory, this should apply only to genes that are expressed exclusively in one condition, although experimental evidence suggests that a broader set of condition-biased genes might follow such a pattern^24,49^. If the hypothesis holds true, it might be very relevant for the evolution of SB genes in *S*. *aurata*. In sequential hermaphrodites, sex ratio is highly unbalanced toward the “first” functional sex, whereas very high variance in reproductive success (Vk) is expected in the “second” functional sex^50^. In protandric hermaphrodites, such as *S*. *aurata*, there are more male breeders, whereas female Vk is much higher^50^, thus making male Ne substantially greater than female Ne^51^. As a consequence, purifying selection might be more effective on male-biased than female-biased genes, explaining the lower dN/dS values that were consistently observed for genes highly expressed in the testis and the faster evolution in Fem-SP6 and Fem-FC3. In protogynous hermaphrodites, the situation should be reversed, with a higher bias between male and female Ne, in favor of the latter^50^. We therefore expect that in protogynous hermaphrodites, male-biased genes evolve much faster than female-biased genes, a hypothesis that can be tested in other fish species.

In conclusion, sequencing and comparative analysis of the *S*. *aurata* genome and transcriptome revealed that SB genes were similar in number and function as in gonochoristic species, but unexpectedly they did not evolve more rapidly than UB genes. This might be due to stronger functional constraints on sequence evolution posed by the observed greater pleiotropy in *S*. *aurata* SB genes, a hypothesis that was supported by the identification of more than 9000 conserved non-coding elements unique to the genomes of three sequential hermaphrodite fish. Likewise unexpected was the observation that both synonymous and non-synonymous evolutionary rates were higher in female-biased genes that have either a strong expression bias (log_2_ FC > 3) or a female-specific expression pattern across diverse fish lineages. These results are most likely a consequence of weaker purifying selection on genes expressed either predominantly or exclusively in females. In *S*. *aurata*, fewer individuals reproduce as females and they have high reproductive variance. This leads to a much lower Ne for females and consequently less effective negative selection. The evolution of SB genes in a sequential hermaphrodite therefore appears to be highly divergent from what observed in gonochoristic species.

## Methods

### Production of sea bream mitogynogenetic diploids

*S*. *aurata* mitogynogenetic double haploid production was carried out in February 2013 at the hatchery of Valle Cà Zuliani srl (Monfalcone, Gorizia, Italy) by following the procedure described below.

Gametes were collected from sea bream breeders induced to spawn by photo-thermo period manipulation for fingerling commercial production and two- three ovulating females were rapidly netted, anesthetized, and stripped. Oocytes to be manipulated (4–60 mL for female) were selected by checking quality on the basis of buoyancy and morphology. Sperm was taken immediately after egg collection from at least three males and maintained at 2–4 °C until use. Double haploid production was obtained adapting the protocol described in ref. ^52^. In brief, about 30–40 mL of eggs were fertilized with UV-irradiated (254 nm; 3300 erg mm^2^) sperm, mixing 1 part of eggs, 3 parts of seawater, and 1 part of sperm diluted 1:100 with extender (375 mOsm kg^−1^ KCl, pH 7.4). A total of 5–10 mL of eggs were fertilized with intact sperm and the appearance of the first cleavage was monitored. This allowed to obtain normal diploid controls. The haploid chromosome set was then doubled by applying a pressure shock at 80 MPa for 4 min at the time of the onset of the first cleavage (76–84 min after fertilization), on the basis of the preliminary fertilization. Motility in intact and UV-irradiated sperm was checked before fertilization. In order to evaluate sperm irradiation efficiency, haploid controls were obtained from not shocked eggs. Eggs were incubated at 18 °C until hatching and larval rearing was classically conducted until larva depletion (40 days post hatching). Mitogynogenetic diploid larvae were stored in RNAlater and fin clip tissue of mothers preserved in 80% ethanol.

### Genomic libraries preparation and sequencing

DNA extraction was carried out from an entire mitogynogenetic double haploid 39 days post-hatching larva and from fin clip tissue of the mother using Invisorb® Spin Tissue Mini Kit CE (STRATEC Biomedical AG, Germany). Samples were treated with RNAase and eluted in 200 µl Elution Buffer provided with the kit. DNA quantity and quality were assessed using a Qubit®ds DNABR Assay Kit (Invitrogen–Thermo Fisher Scientific, Waltham, Massachusetts, USA), NanoDrop ND-1000 spectrophotometer (Thermo Fisher Scientific), and by loading an aliquot on TAE 1 × 0.8% agarose gel.

Homozygosity of the mitogynogenetic double haploid 39 days post-hatching larva (i.e., sample 199) was analyzed with 18 microsatellite markers (Fd79, Gd64, BId18, CId32, CId44, Dd84, Ed02, DId19, FId57, CId38, FId15II, FId31II, CId29, FId56, CId14, DId70, BId39, and CId65). The sea bream microsatellite loci were selected for their position on a first generation linkage map^53^, later improved in the context of a quantitative trait loci analysis on the same species^54^. All the 18 loci belong to 5 different linkage groups (LG3, LG5, LG7, LG14, and LG15) and for LGs with more than one locus, selection was made in order to cover most of each LG’s length (see Supplementary Table 5).

Multiplex (5plex or 4plex) PCR amplifications of microsatellite loci were performed under the following conditions: a final volume of 20 µl reaction mixture contained 0.8 units of Taq DNA Polymerase (Promega), 1 × Thermophilic DNA Polymerase buffer (magnesium free), 1 mM MgCl_2_, 70 µM dNTPs, 2–3 pmol of primer according to amplification efficacy, and 10 ng of genomic DNA. The thermal profile included the following: (i) a predenaturation step of 3 min at 94 °C; (ii) 33 cycles consisting of 1 min at 94 °C, 50 s at 54 °C or 58 °C, and 1 min at 72 °C; (iii) a final step of 5 min at 72 °C. Forward primers were fluorescently labeled using 6-FAM, HEX, or TAMRA. Up to nine different loci were pooled and sequenced on an ABI3730XL capillary analyzer (Applied Biosystems, Foster City, CA, USA) with size standard Rox 400 (Applied Biosystems) at Macrogen, Inc. (Korea). Genotyping was carried out visualizing sequencing results with the software STRand 2.2.30^55^ (http://www.vgl.ucdavis.edu/STRand). Length calls were manually given for all loci, in order to avoid scoring errors due to automatic rounding of effective length scores.

In the mother (sample 197) of the mitogynogenetic diploid larva, out of the 18 loci analyzed, 12 were polymorphic and thus informative for the issue addressed (BId18, CId44, Dd84, FId57, CId38, CId29, FId56, CId14, DId70, BId39, CId65, and FId15II). For one of these (FId15II), the presence of a null allele was detected. Being the other six loci monomorphic in the female, they were not usable to detect allele segregation in the progeny. The results observed for the 12 informative loci were consistent with the hypothesis that the 39 dph larva generated is a mitogynogenetic diploid, being its genotype homozygous for all these loci (see Supplementary Table 6).

The DNA isolated from the double haploid offspring larva and her maternal parent were used to construct two different shotgun libraries (insert length: 180–200 bp) by following the standard protocol of the TruSeq DNA sample preparation kit (Illumina, CA, USA). These two libraries were sequenced on an Illumina HiSeq 1000 instrument following a 100 PE strategy.

In order to help the genome scaffolding different strategies were implemented. A mate-pair library of the same female mentioned above was synthesized by following the standard protocol provided in the Nextera Mate pair library preparation kit (Illumina). Then, a size selection step was performed to retain only fragments with a length of 3–6 kb. This mate-pair library was sequenced on an Illumina HiSeq 1000 instrument following a 100 PE strategy. A shotgun library (insert length > 500 bp) of the double haploid offspring was constructed following the protocol of the TruSeq DNA sample preparation kit and sequenced on both Illumina HiSeq (100PE) and a MiSeq (300PE) instruments. High-molecular-weight DNA was isolated using a Genomic-tip 100/G (Qiagen) and a PacBio library construction kit and sequencing (eight SMRT cells) was performed by Cold Spring Harbor Laboratory (Cold Spring Harbor, NY, USA).

### De novo assembly and scaffolding

Raw Illumina reads were deposited in the Sequence Read Archive (SRA) repository under the accession numbers reported in Supplementary Data 9. Reads were initially assembled with CLC Genomics Workbench 10 (www.qiagenbioinformatics.com) using default parameters and a Linux cluster (24 cores and 254  GB of RAM). Scaffolds below 500 bp were removed, as they are of limited use and probably artifacts. A BLASTN search^56^ (*e*-value threshold = 1 × 10^−06^) against the complete *S*. *aurata* mitochondrial genome (GenBank: LK022698.1) was performed to find and delete mitochondrial contamination from the assembly.

PacBio long reads were used to fill in gaps and join scaffolds with the software PbJelly2, a highly automated pipeline that aligns long sequencing reads to high-confidence draft assembles^57^. A further scaffolding step was performed with SSPACE_standard (− *k* = 5)^58^ using 83,018 published Bacterial Artificial Chromosome (BAC) sequences^59^ and 1.98 M mate-pair reads.

### Improving assembly using Linkage maps

After assembly and scaffolding, quality and contiguity of the first genome draft was further improved by using a high-throughput linkage mapping approach. Three high-density linkage maps with 11,572^60^, 14,481, and 14,506 (Aslam ML, personal communication) single-nucleotide polymorphism (SNP) markers mapped against the draft genome were used to join and orientate scaffolds. In total, 92% of markers were anchored to the genome of which 64.2% were oriented and the remaining 8% was unplaced. The mapped whole-genome sequencing scaffolds were then ordered and oriented using ALLMAPS^61^. Scaffolds with only one SNP kept their original orientation. To obtain genome assembly statistics, contigs shorter than 500 bp were removed, and Assemblathon 2 script was used^62^. Contig break was set to 25 bp.

### Assessment of protein completeness

To provide measures for quantitative assessment of the genome assembly, a Benchmarking Universal Single-Copy Orthologs (BUSCO v.3)^63^ analysis was performed, based on an evolutionarily informed expectation of gene content. The Actinopterygii dataset, containing 4584 well-conserved genes, was employed to investigate the completeness of the assembly.

### Genome masking

Repetitive elements were identified using RepeatModeler (http://www.repeatmasker.org/RepeatModeler/) and used to search against a fish protein database without transposon proteins to ensure exclusion of gene fragments. The search was performed using BLAST program^56^ and setting an *e*-value cutoff of 1 × 10^−10^. Sequences with a match to genes were removed along with 50 bp upstream and downstream of the BLAST hit using the program ProtExcluder available at http://www.hrt.msu.edu/uploads/535/78637/ProtExcluder1.2.tar.gz. If the remaining sequences were shorter than 50 bp, the entire sequence was excluded. After this filtering step, 852 different repetitive elements were identified and used to mask the *S*. *aurata* genome using RepeatMasker (http://www.repeatmasker.org). We were able to mask 20.54% of the genome.

### Gene prediction

Gene prediction considered several sources of evidence such as RNA-seq, nucleotides and proteins alignments, de-novo gene training and prediction.

As far as RNA-seq data, a total of 23 libraries coming from different tissues were used for gene prediction. Adapters clipping and quality trimming were performed using Timmomatic software^64^. Trimmomatic was run setting an average minimum quality score of 20 within a sliding window of 5. The minimum read length was set to 35 bp. RNA-seq reads were aligned on the reference genome using GSNAP program^65^ using default parameters and enabling the detection and alignment of spliced reads. Supplementary Table 7 reports trimming and alignment statistics. Genome-guided transcript reconstruction was performed using StringTie, setting the minimum junction coverage to 3 (option –j)^66^. The transcript reconstruction was performed independently on each single sample. The final redundant dataset contained 1,357,242 transcripts. The transcripts were further assembled using PASA software^67^. PASA, acronym for Program to Assemble Spliced Alignments, is a eukaryotic genome annotation tool that exploits spliced alignments of expressed transcript sequences to automatically model gene structures. PASA produced a final assembly of 241,563 different transcripts.

Nucleotide and proteins sequences belonging to teleost were downloaded from NCBI and aligned to the reference genome using exonerate program (https://www.ebi.ac.uk/about/vertebrate-genomics/software/exonerate). In order to have high-quality alignment, high stringent criteria were used to filter the alignments: 30% identity and 70% alignment coverage at protein level, 50% identity, and 70% alignment coverage at nucleotide level.

Ab-initio de-novo gene training and prediction was performed using five different programs: Augustus^68,69^, Snap^70^, GeneId^71^, Glimmer^72^, and GeneMark^73^. GeneMark was run providing introns coordinates from RNA-Seq read alignments. GeneId was run using tetraodon gene model. Snap, Glimmer, and Augustus were trained using the gene models generated by PASA. Briefly, the PASA alignment assemblies were used to automatically extract protein-coding regions for generating a high-quality dataset for training ab-initio gene predictors. The list of putative gene model generated by PASA were filtered considering only complete genes and validated through a similarity search with blast against a dataset of teleost proteins. Only proteins with a match with an *e*-value lower that 1 × 10^−30^ and an alignment coverage higher than 90% were retained to train the ab-initio predictors.

The previous collected evidence were combined using EvidenceModeler (EVM) program^67^, in order to obtain the single gene model. The EVM software combines ab-initio gene predictions and protein and transcript alignments into weighted consensus gene structures.

EVM prediction produced a total of 50,047 genes. These genes were filtered in order to reduce the number of false positive and low-quality predictions. Three criteria were applied for the filtering. First, genes predicted only by ab-initio programs were considered good only if confirmed by at least four different ab-initio programs, if they were complete (with a start and a stop codon) and longer more than 300 bp. Second, genes supported only by external evidence (e.g., proteins/RNA-seq) need to be confirmed by at least two different evidence or by one external evidence and at least three different ab-initio gene predictors. Third, predicted genes with a low ab-initio support (step 1) were further processed. Genes supported by < 4 ab-initio programs were search against a database of teleost protein sequences. Proteins with a sequence coverage match higher than 70% and an *e*-value lower that 1 × 10^−20^ were recovered.

The gene models passing these filters were further process with PASA in order to predict alternative spliced isoforms and to add the UTR regions. Statistics, calculated using Eval package^74^, of the final prediction in comparison with the sea bass gene prediction are reported in Supplementary Table 3.

Similarity searches of *S*. *aurata* predicted genes were performed against the non-redundant protein database (NCBI) using a BLASTP^56^ with an *e*-value threshold of 1 × 10^−05^. Conserved protein domains and functional annotation were obtained using InterProscan 5^75^ searches against the databases PROSITE patterns, PRINTS, PFAM, PRODOM, SMART, TIGRFAM, and PANTHER. GO and Kyoto Encyclopedia of Genes and Genomes classifications were predicted running BLAST2GO 2.6.0^76^ on the BLAST^56^ and InterProscan outputs.

The *S*. *aurata* genome can be accessed at http://biocluster.her.hcmr.gr/myGenomeBrowser?portalname = Saurata_v1 built using the JBrowse-based MyGenomeBrowser software^77^. The Genome browser allows several options for data mining and retrieval (Gene annotations/sequences, SNP locations). A local BLAST search is also implemented.

### Homology analysis

Homology relationships between *S*. *aurata* and model teleost genomes were reconstructed using OMA v.2.1.1^17^. Pre-computed orthology assignments for the teleost taxonomy group Neopterygii (spotted gar, *Lepisosteus oculatus*, and all teleosts available in Ensembl.org: *Astyanax mexicanus*, *D. rerio*, *Gadus morhua*, *G*. *aculeatus*, *O*. *niloticus*, *Oryzias latipes*, *Poecilia formosa*, *Takifugu rubripes*, *Tetraodon nigroviridis*, and *Xiphophorus maculatus*) were downloaded from the OMA genome browser using the export All-All function. The proteomes of *S*. *aurata* along with the closely related proteomes of the European sea bass *D*. *labrax*^78^ (downloaded from http://seabass.mpipz.mpg.de/cgi-bin/hgGateway) and large yellow croaker, *Larimichthys crocea*^79^ were added in the analysis. Finally, OMA orthology groups were calculated for each species pair using default parameters.

### Phylogenomic analysis

To infer the phylogenetic relationship of *S*. *aurata* and model teleosts, we selected genes that had a one-to-one orthology relationship, thus allowing for a maximum of one species with no ortholog. This filtering resulted in 2032 orthologous groups. The sequences of each orthologous group were aligned using mafft v7.050b (--auto)^80^. The aligned groups were further filtered for sites with more than 30% missing data and concatenated to a matrix using FASconCAT v1^81^. The matrix included 1,097,404 amino acids in total. To exclude divergent and ambiguously aligned regions in the matrix we used Gblocks 0.91b^82^ (parameters -b1 = 9 -b2 = 13 -b3 = 8 -b4 = 10 -b5 = n –p = y), which restricted the alignment to 762,730 high-quality amino acid sites. This dataset was used as the input in RaxML v. 8.0.23^83^. For the phylogenetic reconstruction, first we ran the script *ProteinModelSelection*.*pl* included in RaxML, which selected JTT + F + Γ as the best model. Then, we ran the phylogenetic analysis using the selected model and conducted 100 bootstrap resampling to obtain the branches support. Spotted gar was used as outgroup. The tree was visualized in Dendroscope^84^.

### Gene family expansions/contractions

To study the gene gain and loss of *S*. *aurata* genome compared with other fish species, we used the CAFE v.4 pipeline^85^. We included all species used for the phylogenomic reconstruction apart from the yellow croaker due to the lack of information on gene isoforms. All other species were used in an all-against-all BLASTP search^56^ including only the longest isoform per gene (*e*-value 1 × 10^−05^). BLAST was performed in parallel using the ParaNoblast pipeline^86^ (https://github.com/jacqueslagnel/ParaNoBLast). Then, sequences were clustered using MCL^87,88^ following the software instructions and using the python scripts in CAFE (https://iu.app.box.com/v/cafetutorial-files). Using the recovered phylogeny and an estimate of divergence time for *G*. *aculeatus* and *O*. *niloticus* from TIMETREE (http://www.timetree.org/), we produced an ultrametric tree using r8s^89^. Finally, after the data infile and ultrametric tree preparation, CAFE was run using a significance level threshold for gene family expansions and contractions of 0.01. Large families (> 100 members in any species) were analysed separately following the software instructions. All homology, phylogenomic, and gene family expansion/contraction analyses were run on the IMBBC HPC cluster, HCMR, Heraklion, Greece.

### Conserved non-coding elements

LastDB and Lastall commands from Last aligner^90^ were used to find conserved regions (including CDS) via whole-genome pairwise alignments of the following species: *S*. *aurata*, *G*. *aculeatus*, *O*. *niloticus*, *O*. *latipes, L. calcarifer*, *M. albus*, and *Kryptolebias marmoratus*. All soft-masked genomes and respective annotation files were downloaded either from ensembl.org or from NCBI genomic repositories (available versions on June 2017).

All pairwise genomes alignments were combined into two whole-genome multiple sequence alignments using TBA v12 plus Multiz v11.2 programs:^91^ one containing the multiple sequence alignments of all species and one containing the multiple sequence alignments of the sequential hermaphrodite fishes, respectively, based on the following phylogenetic relationship tree in the modified Newick format, ((*S*. *aurata G*. *aculaetus*) ((*O*. *niloticus* (*K*. *marmoratus O*. *latypes*)) (*L*. *calcarifer M*. *albus*))), and (*S*. *aurata* (*L*. *calcarifer M*. *albus*)).

For removal of coding elements from the conserved regions identified in the multiple sequence alignment blocks, coding regions of each species were retrieved and soft-masked. Additional multiple sequence alignment block filtering was performed to select alignment blocks bigger than 200 columns (bp) and with a minimum absolute identity of 80%. The pipeline available in^92^ was used to identify CNEs.

*S. aurata* genomic coordinates related to CNEs were retrieved from the multiple aligned blocks as two lists: one reporting the CNEs found in all species and one reporting the CNEs found in sequential hermaphrodite species. These two CNE lists were then cross-queried using the GenomicRange R package^93^ to identify non-overlapping CNEs specific for the sequential hermaphrodites. A similar overlapping approach to that outlined above, using the genomic coordinates was then performed to cross-reference the identified CNEs with the transcription start site boundaries within an upstream and downstream 10 Kbp range.

### RNA-seq library preparation and sequencing

The fish used for the transcriptomic experiments were from a broodstock of cultured origin held at the Institute of Marine Biology, Biotechnology, and Aquaculture (Heraklion, Greece). Eight mature fish (four 3-year-old males and four 6-year-old females) were killed by immersion in an ice slurry. The gonad (ovarian or testicular portion depending on the phenotypic sex) and brain tissues were immediately dissected in sterile and RNase-free conditions and stored in RNAlater (Applied Biosystems) at 4 °C overnight and then transferred to − 80 °C until further processing.

Tissues (whole brains, testis, and a section of the ovary, due to their large size) were grinded in liquid nitrogen using pestle and mortar, homogenized in TRIzol® reagent (Invitrogen) and total RNA was extracted from the TRIzol® homogenate according to manufacturer’s instructions. Total RNA was quantified with NanoDrop® ND-1000 (Thermo Scientific) and quality assessed with 2100 Bioanalyzer (Agilent Technologies). All samples had an RNA Integrity Number value > 8.

Finally, 14 samples (3 female brains, 3 male brains, 4 testis, and 4 ovaries) were used to construct mRNA paired-end libraries using the Illumina TruSeq RNA Sample Preparation Kits v2 and following the manufacturer’s protocol (poly-A mRNA isolation with oligo-dT beads, mRNA fragmentation, followed by transcription of first-strand cDNA using reverse transcriptase and random hexamer primers) and sequenced as 150 bp paired reads in one lane of a HiSeq 2500 following the protocols of Illumina, Inc. (San Diego, CA) at the Mr DNA facilities (TX, USA).

### Differential expression analysis

The paired reads of each sample were mapped against the newly assembled genome by means of the STAR aligner^94^ and following the *two-pass* mapping mode. The maximum number of mismatches allowed was set to 10 and only uniquely mapped reads were counted. Read counts for each sample, at the gene level, were extracted by setting the *GeneCounts* quantification while running STAR. Extracted read counts were used for the analysis of differential gene expression and was conducted in EdgeR^95^. Samples were grouped according to sex and expression level was compared for each tissue separately. Genes showing a counts per million (cpm) value < 0.5 in more than half of the samples were filtered out. Extracted reads were normalized with the trimmed mean of M-values (TMM) method in EdgeR. After estimating common and tag-wise dispersions, the exact test provided in EdgeR was used to assess differential expressed genes, with a threshold for a significant FC set to > 1 and a FDR set to < 0.05.

A functional interpretation of the lists of significant genes was obtained through enrichment analysis using the Database for Annotation, Visualization, and Integrated Discovery (DAVID) software^96^. Biological process (BP) annotation categories (BP_direct) were used by setting the gene count equal to 2 and the ease value equal to 0.1. As the DAVID database contains functional annotation data for a limited number of species, it was necessary to use the *D. rerio* Ensembl gene ID corresponding to the *S*. *aurata* homolog gene.

In order to compare SB gene expression across different species, additional RNA sequencing datasets were retrieved from the SRA. Brain and gonad sequenced libraries for both male and female individuals of *D*. *puntazzo*, *E*. *cyanostictus*, *A*. *burtoni*, *O*. *ventralis*, and *J*. *ornatus* were downloaded. Taxonomic family, tissues, number of samples, SRA numbers, and references for each species are included in Supplementary Data 9. *D*. *puntazzo* 100PE reads were mapped against the *S. aurata* genome by means of a STAR *two-pass* mode^94^, whereas the cichlids 50SE RNA-seq reads were mapped against the Ensembl *O*. *niloticus* genome (release 1.0.89). To take into account the fact that mapping was performed against close phylogenetic species instead of the species-specific genomes (which are not available) and read length was different, mismatch counts were set to 15 and 5 for *D*. *puntazzo* and the cichlids, respectively.

To perform the comparative transcriptomic analysis, strictly one-to-one orthology relationships between *S*. *aurata* and *O*. *niloticus* were retrieved with OMA, as previously described, and used as input for the statistical analysis.

Raw reads were normalized as described above and after estimating common, trend and tag-wise dispersions, the likelihood ratio test provided in EdgeR^95^ was used to identify SB genes (i.e., log_2_ FC > 1 and FDR < 0.05) across the target species.

In order to visualize the level of similarity of individual samples of the two datasets (gonads and brains), a Hierarchical Clustering Analysis (HCL) was conducted in WebMeV (http://mev.tm4.org) using log_2_ cpm of TMM-normalized data.

Based on gene expression and comparative analyses several gene datasets were constructed for evolutionary analyses. A first dataset contained genes showing differential expression in *S*. *aurata* gonads (SB, further distinguished in Male-biased and Fem-biased). A second dataset included genes showing strong differential expression (i.e., log_2_ FC ≥ 3) in *S*. *aurata* gonads (Fem-FC3 and Male-FC3). A third dataset contained genes male- or female-biased in all species used for comparative analysis (Fem-6-SP and Male-6-SP). A fourth dataset represented genes showing no differential expression in *S. aurata* gonads (UB).

### Expression breath and plasticity

Tissue specificity (i.e., expression breath) of genes was estimated using the *τ*-index^37^. The index *τ* ranges from 0 to 1 and it is defined as: *τ* = ∑Ni = 1(1 − xi)/*N* − 1, where *N* is the number of tissues and *xi* is the expression profile component normalized by the maximal component value (*τ* = 1 single tissue expression, *τ* = 0 ubiquitous expression). Tissue specificity was calculated on the mean normalized expression (TMM-normalized log_2_ cpm) evaluated in whole larvae (three stages), ovary, testis, brain, gut, heart, liver, skeletal muscle, and spleen. Gene expression data of three different larval stages was obtained from^41^ (only samples kept at control conditions were employed) while gene expression for each tissue was calculated from the RNA-seq data produced in the present study (see Supplementary Data 9).

Gene expression plasticity, estimated by the plasticity index *π*^40^, was quantified across larvae at three stages of development exposed to chronic stress^41^ (Supplementary Data 9) for a total of three pairwise comparisons (control vs. stress-exposed at each stage of development).

Median values of *τ* and *π* were calculated on previously defined groups of genes with specific expression patterns in *S*. *aurata*: SB, Fem-FC3, Male-FC3, Fem-6-SP, Male-6-SP, and UB.

### Synonymous and non-synonymous substitution rates

To estimate the evolutionary rates of SB gene sequences, we downloaded protein-coding sequences of strictly one-to-one orthologs obtained by running OMA (see above) on *S. aurata*, *D*. *labrax*, and *G*. *aculeatus*, and retained the longest transcript for each gene for this analysis. *D*. *labrax* was chosen, because it is the closest teleost species with a high-quality genome, whereas the *G*. *aculeatus* genome is the best annotated, of a closely related outgroup species. A total of 11,855 orthologs were identified and aligned using PRANK (v. 140603)^97^ at the codon level (-*codon*). Then, SWAMP (v. 31-03-14) was used to filter regions with poor alignment with a cutoff of 4 in a window size of 5 and a minimum length of 75 bp^98^. All positions having gaps and *N* were removed from the alignments. We also excluded from analysis alignments shorter than 100 bp. Using this approach, a total of 11,514 one-to-one orthologs were retained.

Lineage-specific evolutionary rates dN (number of non-synonymous substitutions per non-synonymous sites), dS (number of synonymous substitutions per synonymous sites), and their ratio dN/dS were calculated using CODEML in the PAML 4.7 package (*runmode* = 0 and *model* = 1)^99^. Genes with saturated synonymous substitution values dS > 2 or N*dN or S*dS < 1 in the *S*. *aurata* lineage were excluded from the analysis, resulting in a final dataset of 10,783 orthologs.

Median values of dN, dS, and dN/dS were calculated for the same groups of genes defined above: SB, Fem-FC3, Male-FC3, Fem-6-SP, Male-6-SP, and UB. In order to assess the significance of inter-group substitution rate differences, pairwise Wilcoxon’s rank-sum tests were implemented in R.

To estimate the evolutionary rates of tilapia SB gene sequences, we followed the same approach described above. One-to-one *O*. *niloticus*, *O*. *latipes* (as sister species), and *G*. *aculeatus* (as outgroup species) orthologs were aligned. A final dataset of 12,155 orthologs was obtained.

### SNPs analysis as genetic resources

*S. aurata* intraspecific polymorphisms were assessed in a cohort of 357 individuals (see Supplementary Data 9). For 292 individuals, sequencing data belonged to 2b-RAD libraries constructed as reported by^60^. The RAD libraries were representative of three *S*. *aurata* broodstocks with different genetic background. The first dataset (*n* = 67) was provided by the fish farm Valle Ca’ Zuliani (Monfalcone, Italy) and it included fish obtained from the wild (Western Mediterranean and Adriatic sea). The second dataset (*n* = 108) was provided by Ferme Marine du Douhet (La Brée-les-Bains, France). The third dataset (*n* = 117) was provided by Andromeda Group (Greece)^100^.

For the remaining 65 individuals, sequencing data belonged to the RNA-seq libraries (brain and gonads, see Supplementary Data 9) produced in the present study and to the RNA-seq study (larvae, *n* = 51) by^41^ (see Supplementary Data 9). All the SNPs identified were deposited into the Dryad Data Repository^101^ and can be also accessed at http://biocluster.her.hcmr.gr/myGenomeBrowser?portalname = Saurata_v1.

Mapping of reads against the *S*. *aurata* genome was performed by means of BWA samse^102^ and STAR two-pass mode for 2b-RAD and RNA-seq libraries, respectively. SNP discovery and genotyping across all samples was performed simultaneously using standard hard filtering parameters according to GATK (v 3.7) Best Practices; loci with individual read depth below 6 were set to no-call.

A total of 822,426 SNPs were functionally annotated with ANNOVAR^103^. In order to retrieve exonic synonymous and non-synonymous single-nucleotide variations; those leading to stop-codon gain or loss were removed from the analysis.

The number of non-synonymous substitutions (Pn) and synonymous substitution (Ps) were extracted for each gene belonging to groups SB, Fem-FC3, Male-FC3, Fem-6-SP, Male-6-SP, and UB. For the same genes the DoS index and the NI (NI_TG_) as proposed by Stoletzki and Eyre-Walker^47^ were also calculated. For each group, median values of Pn, Ps, DoS, and NI_TG_ were calculated. To assess the significance of between-groups differences, pairwise Wilcoxon’s rank-sum tests were implemented in R.

### Distribution of SB genes across the *S. aurata* genome

Two statistical approaches were used to test whether the distribution of SB genes across the 24 *S*. *aurata* super-scaffolds (i.e., chromosomes) showed any evidence of clustering. A Markov chain model of transition probabilities (described in Supplementary Figure 2) was performed producing as the odds score the comparison between the observed model for each super-scaffold and the model based on the null hypothesis (i.e., independence of gene distribution). The higher this score, the higher the probability of SB genes to be clustered. A Fisher’s exact test was also employed to calculate the probability of finding clustered SB genes compared with the expected distribution.

### Ethics statement

No specific permits were required for the work described here. Animals included in the present study were not subjected to any experimental manipulation. The study was performed in accordance with the EU directive 2010/63/EU and Italian DL 2014/26. Experiments and killing procedures were monitored and carried out by authorized staff to minimize animals’ suffering

### Data availability

Sequence data that support the findings of this study have been deposited in NCBI Short Reads Archive (SRA). Genomic sequences can be found under the following accession numbers: SRR6244977-SRR6244982. Transcriptomic sequences can be retrieved under the following accession numbers: SRR6244977-SRR6244982, SRR6223527-SRR6223532, SRR6223535-SRR6223542, and SRR6237494-SRR6237500. Details are reported in Supplementary Data 9. The *S*. *aurata* genome can be accessed at http://biocluster.her.hcmr.gr/myGenomeBrowser?portalname = Saurata_v1. This Whole Genome Shotgun project has been also deposited at DDBJ/ENA/GenBank under the accession PQWN00000000. All the SNPs identified were deposited into the Dryad Data Repository(10.5061/dryad.cd55md1)^101^.

## Electronic supplementary material

Supplementary information

Description of additional supplementary items

Supplementary Data 1

Supplementary Data 2

Supplementary Data 3

Supplementary Data 4

Supplementary Data 5

Supplementary Data 6

Supplementary Data 7

Supplementary Data 8

Supplementary Data 9
