# Supplementary material for: Genomic analysis of Sparus aurata reveals the evolutionary dynamics of sex-biased genes in a sequential hermaphrodite fish
Source: Commun Biol. 2018 Aug 17;1:119. doi: 10.1038/s42003-018-0122-7 (PMC6123679; doi:10.1038/s42003-018-0122-7)
Supplement: Supplementary file 1 — Supplementary information [file 42003_2018_122_MOESM1_ESM.pdf]

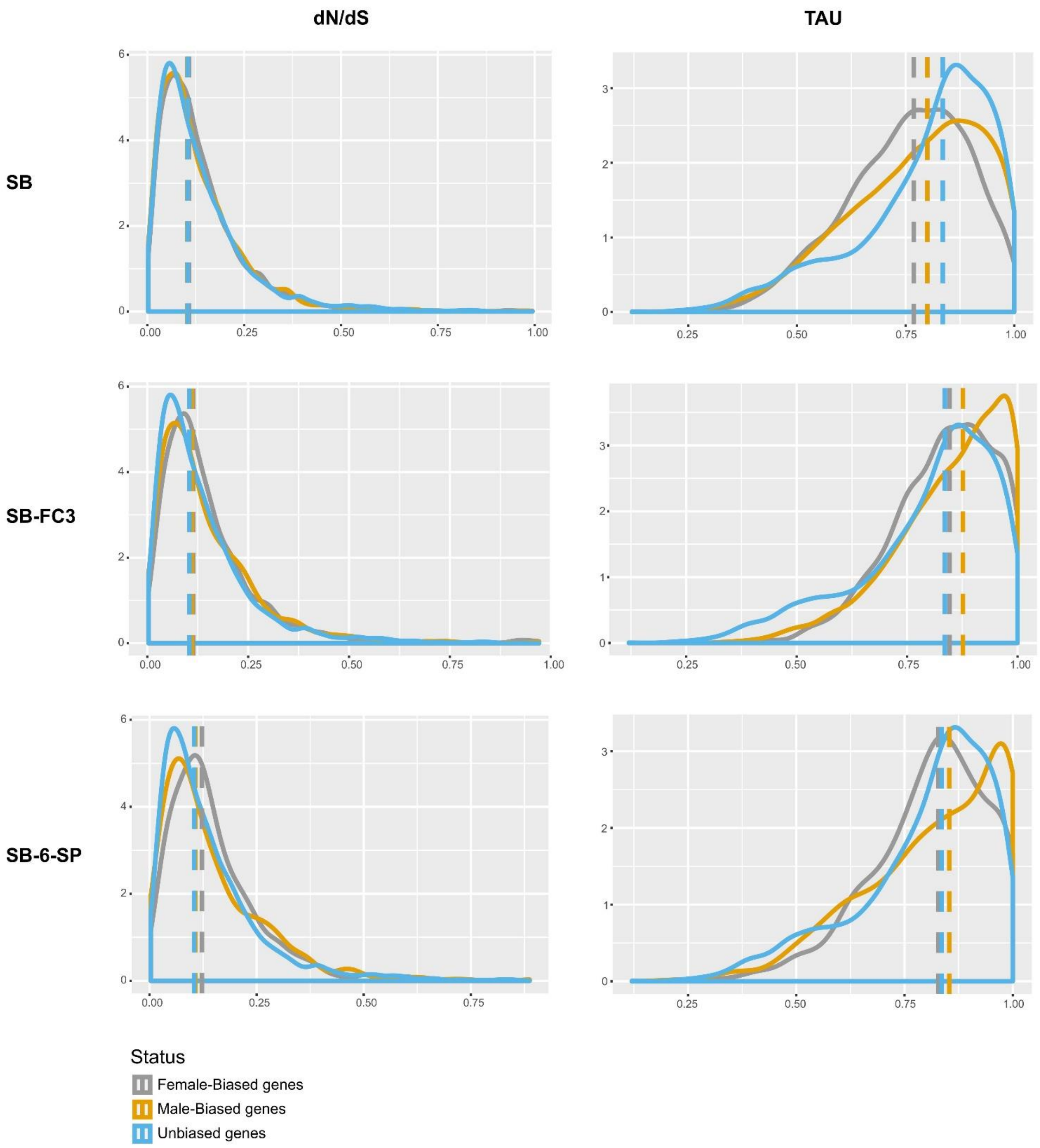

Supplementary Figure 1. Specificity. Density functions of dN/dS and τ values of sex-biased (SB) gene sets compared to those of unbiased genes. SB: sex-biased genes; SB-FC3: sex-biased genes $\log_2$ FC ≥ 3; sex-biased genes in all species used for comparative analysis (*i.e.* sparids and cichlids).

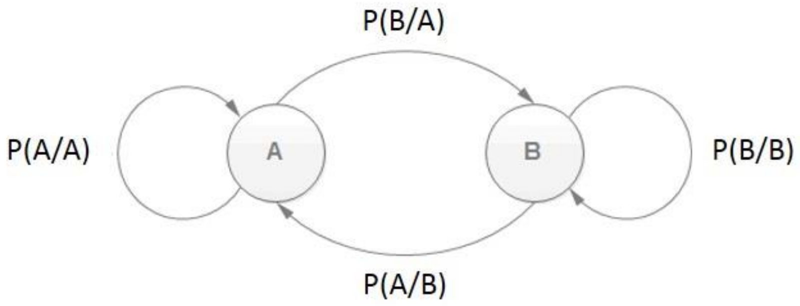

A = sex-biased genes (SB)
B = unbiased genes

Arrows = possible transitions, each labeled with a transition probability P.

NULL MODEL

P(A/A) ≈ P(A/B) ≈ f(A)
P(B/A) ≈ P(B/B) ≈ f(B)

$$P\ (\text{NULL-model}) = P(A)^{nA} * P(B)^{nB}$$

GIVEN A CHROMOSOME (chr)

$$\mathbf{P\ (chr|model)} = \prod_{i=1}^{L-1} P(gene_{i+1}\ /\ gene_{i})$$

To compare the models, the odds ratio will be:

$$\frac{\mathbf{P\ (chr|model)}}{\mathbf{P\ (chr|NULL)}}$$

Supplementary Figure 2. Markov chain model of transition probabilities. Graphical representation and description of the Markov chain model of transition probabilities applied for genes clustering analysis on sea bream chromosomes.

Supplementary Table 1. Statistics. Genome assembly statistics (contigs and scaffolds).

Number of scaffolds	55 202
Total size of scaffolds	763 166 337
Longest scaffold	350 738
Shortest scaffold	932
Number of scaffolds > 1K n	55 179 (100%)
Number of scaffolds > 10K n	18 768 (34%)
Number of scaffolds > 100K n	810 (1.5%)
Number of scaffolds > 1M n	0 (0%)
Number of scaffolds > 10M n	0 (0%)
Mean scaffold size	13 825
Median scaffold size	4 414
N50 scaffold length	37 409
L50 scaffold count	5 476
scaffold %A	29.06
scaffold %C	20.86
scaffold %G	20.87
scaffold %T	29.06
scaffold %N	0.15
scaffold %non-ACGTN	0
Number of scaffold non-ACGTN nt	0
Percentage of assembly in scaffolded contigs	33.8%
Percentage of assembly in unscaffolded contigs	66.2%
Average number of contigs per scaffold	1.1
Average length of break (> 25 Ns) between contigs in scaffold	140
Number of contigs	62 363
Number of contigs in scaffolds	18 039
Number of contigs not in scaffolds	44 324
Total size of contigs	762 158 065
Longest contig	350 738
Shortest contig	49
Number of contigs > 1K n	55 964 (89.7%)
Number of contigs > 10K n	19 078 (30.6%)
Number of contigs > 100K n	731 (1.2%)
Number of contigs > 1M n	0 (0%)
Number of contigs > 10M n	0 (0%)
Mean contig size	12 221
Median contig size	3 480
N50 contig length	35 872
L50 contig count	5 750
contig % A	29.1
contig % C	20.89
contig % G	20.89
contig % T	29.1
contig % N	0.02
contig %non-ACGTN	0
Number of contig non-ACGTN nt	0

Supplementary Table 2. BUSCO assessments. Summary of benchmarks of the BUSCO v. 3 assessment. *actinopterygii dataset.

Number of total BUSCO groups searched*	4 584
Complete BUSCOs (%)	90.8
Complete and single copy (%)	88.9
Complete and duplicated (%)	1.9
Fragmented BUSCOs (%)	5.2
Missing (%)	4.0

Supplementary Table 3. Gene prediction statistics.

	**Sea bream**	**Sea bass**
Genes	30,454	26,719
Transcripts	61,850	26,719
Average gene size	11,856	12,978
Coding exons (bp)	7%	6%
Coding average size	168	169
Intron average size	1,045	1,199
5'UTR average size	298	295
3'UTR average size	1,267	1,394

Supplementary Table 4. Gene expression of genes belonging to gene families and single copy genes. The number of genes listed in a gene family and the number of single copy genes found in unbiased, male-biased genes and female-biased were reported, specifying also relative percentages.

	**UNBIASED genes**	**MALE-BIASED genes**	**FEMALE-BIASED genes**
**Family**	11 865	6 920	5 448
**Single-copy**	2 211	1 604	2 406
**Total**	**14 076**	**8 524**	**7 854**
**% Family**	84%	81%	69%
**% Single-copy**	16%	19%	31%

Supplementary Table 5. For each analyzed microsatellite locus, harboring Linkage Group (LG), position across LG in centiMorgans (cM) and the whole LG length were reported.

	**Locus**	**LG**	**Position (cM)**	**LG length (cM)**
1	CId32	3	0.0	33.4
2	CId44	3	7.5	33.4
3	Dd84	3	11.9	33.4
4	Ed02	3	21.1	33.4
5	DId19	3	21.6	33.4
6	Fd79	3	28.3	33.4
7	Gd64	3	33.4	33.4
8	CId29	5	7.3	45.2
9	BId39	5	21.1	45.2
10	DId70	5	45.2	45.2
11	CId38	7	12.6	58.3
12	FId56	7	16.7	58.3
13	CId65	7	58.3	58.3
14	BId18	11	17.1	52.9
15	FId31II	14	52.2	53.2
16	FId57	15	0.0	58.5
17	FId15II	15	10.4	58.5
18	CId14	15	32.2	58.5

Supplementary Table 6. Segregating alleles in the mitogynogenetic diploid larva. Loci in bold are those informative.

**Locus**	**Maternal genotype**	**Larval genotype**
CId32	161/161	
**CId44**	**157/165**	**157**
**Dd84**	**166/192**	**166**
Ed02	216/216	
DId19	219/219	
Fd79	102/102	
Gd64	95/95	
**CId29**	**156/162**	**162**
**BId39**	**212/220**	**220**
**DId70**	**216/222**	**222**
**CId38**	**99/101**	**101**
**FId56**	**162/164**	**162**
**CId65**	**217/223**	**223**
**BId18**	**87/89**	**89**
FId31II	122/122	
**FId57**	**224/236**	**224**
**FId15II**	**122/null**	**122**
**CId14**	**157/159**	**159**

Supplementary Table 7. Statistics of RNA-seq reads preprocessing.

		Number of reads after trimming				
**Sample**	**Number of raw paired reads**	**Reads paired**	**% read paired**	**R1 unpaired**	**R2 unpaired**	**% alignment**
Brain	67,001,969	30,569,884	45.63	35,753,308	159,231	95.41
Gut	60,107,008	28,881,894	48.05	30,013,843	134,710	94.56
Heart	77,170,546	38,640,569	50.07	37,691,927	150,729	95.13
Larvae	70,747,315	33,700,512	47.64	33,372,917	149,276	95.56
Liver	50,350,430	26,736,808	53.10	23,039,779	102,123	96.53
Muscle	43,881,362	23,758,618	54.14	19,737,981	82,884	95.36
Spleen	40,284,941	19,260,672	47.81	20,582,179	91,857	95.68
SaFB1_S14	8,640,440	7,207,200	83.41	1,244,906	99,921	98.82
SaFB2_S15	8,869,530	8,182,389	92.25	514,620	98,887	98.8
SaFB3_S16	10,401,219	9,539,785	91.72	664,477	108,185	98.98
SaFB4_S17	8,845,907	8,113,636	91.72	563,956	93,175	98.95
SaFG1_S22	17,097,428	15,542,910	90.91	1,247,545	164,193	98.82
SaFG2_S23	16,620,614	15,263,627	91.84	1,063,939	153,501	98.49
SaFG3_S24	17,135,858	15,730,597	91.80	1,107,462	156,336	98.75
SaFG4_S25	16,146,718	14,742,661	91.30	1,118,593	130,908	98.79
SaMB1_S10	10,469,426	9,686,374	92.52	587,432	106,386	98.9
SaMB2_S11	14,274,087	13,258,617	92.89	748,375	151,826	99.01
SaMB3_S12	12,904,219	11,811,073	91.53	849,896	135,650	98.91
SaMB4_S13	14,216,055	13,025,753	91.63	918,828	141,623	99.01
SaMG1_S18	16,536,753	15,348,172	92.81	905,504	142,334	99.02
SaMG2_S19	15,741,014	14,580,919	92.63	877,735	152,284	99.04
SaMG3_S20	16,327,351	15,104,414	92.51	929,589	153,173	99.04
SaMG4_S21	15,930,929	14,814,650	92.99	842,578	148,919	99.02
