# Supplementary material for: Genomic analysis of Sparus aurata reveals the evolutionary dynamics of sex-biased genes in a sequential hermaphrodite fish
Source: Commun Biol. 2018 Aug 17;1:119. doi: 10.1038/s42003-018-0122-7 (PMC6123679; doi:10.1038/s42003-018-0122-7)
Supplement: Supplementary file 2 — Description of additional supplementary items [file 42003_2018_122_MOESM2_ESM.pdf]

DESCRIPTION OF ADDITIONAL SUPPLEMENTARY ITEMS

Genomic analysis of *Sparus aurata* reveals the evolutionary dynamics of sex-biased genes in a sequential hermaphrodite fish

Marianna Pauletto, Tereza Manousaki, Serena Ferraresso, Massimiliano Babbucci, Alexandros Tsakogiannis, Bruno Louro, Nicola Vitulo, Viet Ha Quoc, Roberta Carraro, Daniela Bertotto, Rafaella Franch, Francesco Maroso, Muhammad L. Aslam, Anna K. Sonesson, Barbara Simionati, Giorgio Malacrida, Alessandro Cestaro, Stefano Caberlotto, Elena Sarropoulou, Costantinos C.

Luca Bargelloni

Supplementary Data 1. Gene family expansions and contractions. Summary results of gene family expansions and contractions for sea bream and the other teleosts used in the analysis (p-value 0.01). The large families (more than > 100 members in any species) were analyzed separately following the software instructions. Sex-biased genes in sea bream that fall within the extracted-contracted families are given separately.

Supplementary Data 2. Gene families and sex-biased genes. The table reports the two gene families including only male-biased genes and the three gene families containing differentially expressed (DE) genes in both gonad and brain.

Supplementary Data 3. Gene expression analysis. List of the differentially expressed genes between *Sparus aurata* males and females. LogFC, logCPM, PValue and FDR are those obtained from edgeR. Gene annotation has been added. Blue color indicates genes highly expressed in males, while red color indicates genes highly expressed in females.

Supplementary Data 4. Functional enrichment. DAVID functional enrichment analysis performed on male- and female- biased genes. BP category, BP terms, gene counts, p-values, fold enrichments and corrected p-values are reported.

Supplementary Data 5. Genes clustering. Chromosome odds scores and Fisher's p-values calculated on spatial distribution of sex-biased gene sets. "Sea bream DEGs" refers to differentially expressed genes in *Sparus aurata* gonads, while "sea bream orthologous DEGs" refers only to the subset of genes used in evolutionary analyses (i.e. Male-biased, Female-biased, Fem-FC3, Male-FC3, Fem-6-SP, and Male-6-SP).

Supplementary Data 6. Evolutionary rates of *Oreochromis niloticus* sex-biased genes. Median values of dN, dS, and dN/dS were reported for the following groups of genes: male-biased, female-biased, unbiased, male-biased-FC<3, female-biased-FC<3, male-biased-FC>3, female-biased-FC>3. The results of the Wilcoxon rank test assessing the significance of inter-group substitution rate differences were also reported.

Supplementary Data 7. CNEs analysis. List of the CNEs specific of the sequential hermaphrodite fishes within 10K bps upstream or downstream a transcription start site.

Supplementary Data 8. Direction of Selection. Per gene and median DoS values calculated for Female-biased, Male-biased, and Unbiased group of genes. For each group, Ni is also reported.

Supplementary Data 9. Genomic resources. Summary of the genomic sequences produced or employed in the present study.
